# Co-expression network analysis of genes and networks associated with wheat pistillody

**DOI:** 10.7717/peerj.13902

**Published:** 2022-08-24

**Authors:** Zhenyong Chen, Mingli Liao, Zaijun Yang, Weiying Chen, Shuhong Wei, Jian Zou, Zhengsong Peng

**Affiliations:** 1 Key Laboratory of Southwest China Wildlife Resources Conservation (Ministry of Education), College of Life Science, China West Normal University, Nanchong, Sichuan, People’s Republic of China; 2 School of Agricultural Science, Xichang University, Xichang, Sichuan, People’s Republic of China

**Keywords:** Co-expression network analysis, Pistillody development, Wheat, Transcription factor

## Abstract

Crop male sterility has great value in theoretical research and breeding application. HTS-1, whose stamens transformed into pistils or pistil-like structures, is an important male sterility material selecting from Chinese Spring three-pistil (CSTP) wheat. However the molecular mechanism of pistillody development in HTS-1 remains a mystery. RNA-seq data of 11 wheat tissues were obtained from the National Center for Biotechnology Information (NCBI), including the stamens of CSTP and the pistils and pistillodic stamen of HTS-1. The Salmon program was utilized to quantify the gene expression levels of the 11 wheat tissues; and gene quantification results were normalized by transcripts per million (TPM). In total, 58,576 genes were used to construct block-wise network by co-expression networks analysis (WGCNA) R package. We obtained all of modules significantly associated with the 11 wheat tissues. AgriGO V2.0 was used to do Gene Ontology (GO) enrichment analysis; and genes and transcription factors (TFs) in these significant modules about wheat pistillody development were identified from GO enrichment results. Basic local alignment search tool (BLAST) was used to align HTS-1 proteins with the published pistillody-related proteins and TFs. Genes about wheat pistillody development were analyzed and validated by qRT-PCR. The MEturquoise, MEsaddlebrown, MEplum, MEcoral1, MElightsteelblue1, and MEdarkslateblue modules were significantly corelated to pistillodic stamen (correlation *p* < 0.05). Moreover, 206 genes related to carpel development (GO:0048440) or gynoecium development (GO:0048467) were identified only in the MEturquoise module by Gene Ontology (GO) analysis, and 42 of 206 genes were hub genes in MEturquoise module. qRT-PCR results showed that 38 of the 42 hub genes had highly expressed in pistils and pistillodic stamens than in stamens. A total of 15 pistillody development-related proteins were validated by BLAST. Transcription factors (TFs) were also analyzed in the MEturquoise module, and 618 TFs were identified. In total, 56 TFs from 11 families were considered to regulate the development of pistillodic stamen. The co-expression network showed that six of HB and three of BES1 genes were identified in 42 hub genes. This indicated that TFs played important roles in wheat pistillody development. In addition, there were 11 of ethylene-related genes connected with TFs or hub genes, suggesting the important roles of ethylene-related genes in pistillody development. These results provide important insights into the molecular interactions underlying pistillody development.

## Introduction

Common wheat (*Triticum aestivum* L.) is a major source of protein, vitamins, and minerals for humans (Ramírez-González et al., 2018). Although the global wheat output has steadily increased in recent years, some studies showed that wheat yields hardly meet the increasing demand of the human population (The International Wheat Genome Sequencing Consortium, 2014; Marcos-Barbero et al., 2021). Thus, many researchers strive to improve grain productivity and qualities. The development of wheat flowers affects grain yields and qualities (Yang et al., 2015; Ullah et al., 2022). Various wheat flower mutant materials have been reported in the past decade (Murai & Tsunewaki, 1993; Murai et al., 2002; Murai, 2013). Mutant (cr)-N26 and (cr)-CSdt7BS are male sterility materials, whose stamen transforms into pistil or pistil-like structure, a phenomenon called pistillody (Murai & Tsunewaki, 1993; Murai et al., 2002; Murai, 2013). (cr)-N26 and (cr)-CSdt7BS mutants contain the exogenous genes of goat weed; therefore, the pistillody of (cr)-N26 and (cr)-CSdt7BS are caused by heterologous cytoplasmic male sterility (Murai et al., 2002). In 2013, Peng et al. found a new pistillody mutant called HTS-1 from Chinese Spring three-pistil (CSTP) wheat (Peng, 2003; Peng et al., 2013). CSTP is a near-isogenic line of the common wheat variety, Chinese Spring, with the *Pis1* gene derived from the three-pistil mutant (Peng et al., 2013). The genetic base of HTS-1 was different from those of (cr)-N26 and (cr)-CSdt7BS (Murai, 2013; Peng et al., 2013). HTS-1 mutants do not contain an exogenous cytoplasm; therefore, the pistillody of HTS-1 is controlled by nuclear genes (Peng et al., 2013). Previous reports showed that the molecular mechanisms of wheat stamen transformation into pistils were very complex (Murai et al., 2002; Zhu et al., 2008; Yamamoto et al., 2013; Yang et al., 2018). Murai et al. (2002) found that reduced wheat *APETALA3* homolog (*WAP3*) expression could be associated with the induction of pistillody in (cr)-CSdt7BS. The mitochondrial gene *orf25* of *Aegilops crassa* cytoplasm is considered a candidate as the cytoplasmic factor related to pistillody in alloplasmic wheat (Hama et al., 2004). Zhu et al. (2008) reported a mitochondrial gene called *orf260*^*cra*^, which could lead to pistillody in the stamen of (cr)-CSdt7BS. *In situ* hybridization analysis showed that the calmodulin-binding protein gene, WHEAT CALMODULIN-BINDING PROTEIN 1 (*WCBP1*), was highly expressed in pistil-like stamens at early to late developmental stages (Yamamoto et al., 2013). This finding suggests that the *WCBP1* gene plays an important role in pistil-like stamen development (Yamamoto et al., 2013). The homologous transformation of stamens into pistils in HTS-1 florets is determined by the interaction of two recessive karyo genes, *hts1* and *hts2* (Peng et al., 2013). Yang et al. (2015) identified 206 differentially expressed genes (DEGs) correlated to HTS-1 stamen and pistil development using RNA-seq analysis. Among them, 123 genes were down regulated and 83 genes were up regulated. But genes about pistillody development have never been identified in this study. Sun et al. (2019) reported that the EPIDERMAL PATTERNING FACTOR-LIKE (*TaEPFL1*) gene has a higher expression in pistillodic stamens than in pistils and stamens. The heterologous expression of the *TaEPFL1* gene in *Arabidopsis* causes shortened filaments and pedicels, which might induce stamens to transform into pistils or pistil-like structures in wheat. Furthermore, some transcription factors (TFs) of MADS-box (*e.g.*, MCM1 in yeast, AG in *Arabidopsis*, DEFICIENS in *Antirrhinum*, and SRF in mammals) and the DROOPING LEAF (*DL*) gene of the YABBY family regulate the development of wheat pistillodic stamens (Murai, 2013; Hama et al., 2004; Meguro et al., 2003; Ishikawa et al., 2009; Zhang et al., 2015; Wang et al., 2015; Zhu et al., 2015). The expression pattern analysis indicates that *TaDL1*, *TaDL2*, and *TaDL3* might cause the stamen of HTS-1 to completely or partially transform into pistils (Zhang et al., 2015). These researches reported on the development of wheat pistillody. However, the mechanisms of pistillody are still unclear.

With the rapid improvement of high-throughput sequencing and computer analysis technology, complex biological problems can be systematically studied using a variety of high-throughput sequenced data. Various network analysis methods, such as neural network analysis (Greenside et al., 2018), protein–protein interaction network analysis (Stelzl et al., 2005), and WGCNA (Zhang & Horvath, 2005), have been constructed and widely applied to reveal molecular mechanisms using high-throughput sequenced data. WGCNA is an efficient method for studying correlation networks of highly correlated genes (Langfelder & Horvath, 2008). According to gene expression levels in different tissues, genes with similar expression patterns can be clustered into the same module by WGCNA (Zhang & Horvath, 2005). Therefore, WGCNA is widely used to explore the hub genes, module–trait relationships, and gene interaction networks of flower development in various plants (Hollender et al., 2014; Roberts & Roalson, 2017; Ma, Ding & Li, 2017). In particular, Ramírez-González et al. (2018) using 209 RNA-seq samples from 22 tissue types, analyzed the gene expression pattern of wheat Azhurnaya cultivar; and established tissue- and stress-specific co-expression networks that revealed the extensive coordination of homoeolog expression throughout wheat development. These networks, including spikelet development networks, and detailed gene expression atlases were deposited in wheat electronic fluorescent pictographic (eFP) browser (http://bar.utoronto.ca) (Ramírez-González et al., 2018). However, networks in the wheat eFP browser do not reveal and provide information about the mechanisms of genes for pistillody development. The Azhurnaya cultivar is not a pistillodic wheat. Furthermore, the systematic identification of genes associated with pistillody development by WGCNA has not been reported yet.

The present study employed WGCNA to analyze 11 RNA-seq data from wheat tissues, including the normal stamens of CSTP and the pistils and pistillodic stamens of HTS-1, to explore genes related to wheat pistillody development and their interaction mechanisms (Yang et al., 2015). The relationships between modules and specific flower tissues from the wheat RNA-seq data were explored. Then, genes related to pistillody development were identified from the modules that were remarkably associated with pistillody by GO analysis. Quantitative real-time PCR (qRT-PCR) was used to validate the expression pattern of candidate genes related to the development of pistillodic stamens in HTS-1 to validate the WGCNA results. This work provides further insights into the molecular mechanisms underlying wheat pistillody development.

## Materials & Methods

### Plant materials and RNA extraction

CSTP and its pistillody mutant, HTS-1, were planted in the experimental field of China West Normal University, Nanchong, China in the last week of October 2020. The pistils and pistillodic stamens of HTS-1 were collected at the early flowering stage in the middle of February 2021, and the normal stamens of CSTP were harvested at the same time. The three flower materials were collected from 30 HTS-1 and CSTP plants. Each material had two biological replicates. Particularly, only the first florets of the central spikes were collected. The flower materials were immediately frozen with liquid nitrogen and maintained in an ultra-low temperature freezer (Forma 900 Series; Thermo Scientific, Waltham, MA) at −80 °C. The total RNAs of the pistils and pistillodic stamens of HTS-1 and the stamens of CSTP were extracted using a Plant Easy Spin RNA Miniprep Kit (Biomiga, San Diego, CA). DNase treatment was performed before proceeding with complementary DNA (cDNA) synthesis. The quality of the cDNA was determined by gel electrophoresis. cDNA concentration was calculated and adjusted to 500 ng/µL using a spectrophotometer (Thermo, NanoDrop 2000).

### Data source

The following RNA-seq data of common wheat were obtained from the National Center for Biotechnology Information (NCBI) Sequence Read Archive database (SRA, http://www.ncbi.nlm.nih.gov/sra): root (ERR424732), stem (ERR424762), flag leaf (SRR3068439), booting spike (SRR6802610), ear spike (SRR6802611), rachis (SRR6802608), early ovary (SRR6802613), late ovary (SRR6802612), HTS-1 pistil (SRR1175868), HTS-1 pistillodic stamen (SRR1177760), and CSTP stamen (SRR1177761). CSTP is a backcross descendant of Chinese spring and three-pistil wheat, and each CSTP floret has three pistils and three stamens (Peng et al., 2013). HTS-1 is a pistillody mutant from CSTP (Peng et al., 2013). Some HTS-1 stamens transform into pistils or pistil-like structures (Peng et al., 2013). Therefore, the RNA-seq data of HTS-1 pistil (SRR1175868) and CSTP stamen (SRR1177761) are wild types, and HTS-1 pistillodic stamen (SRR1177760) is a wheat flower mutant. All of the RNA-seq data were generated by the Illumina HiSeq series platform, and the layout were in paired end reads. Wheat coding sequences (CDSs) and protein sequences were downloaded from the Ensembl Plants database (version 41, http://ftp.ensemblgenomes.org/pub/plants/release-41/). A total of 133,346 CDSs and protein sequences were obtained.

### Gene quantification analysis

Salmon (Patro et al., 2017) was utilized to quantify the gene expression levels of the 11 wheat tissues mentioned above. First, Salmon index program was employed to create a wheat index using the default parameters, and the 133,346 CDSs of wheat were used as the input data. Next, the 11 RNA-seq data were quantified by the Salmon quant program against the wheat index using the quasi-mapping-based mode. The gene quantification results were normalized by transcripts per million (TPM). The genes in the pistillodic stamens with TPM values less than 0.5 were filtered out to reduce the interference of low expression genes. A total of 58,576 genes remained.

### WGCNA

**Figure 1:**
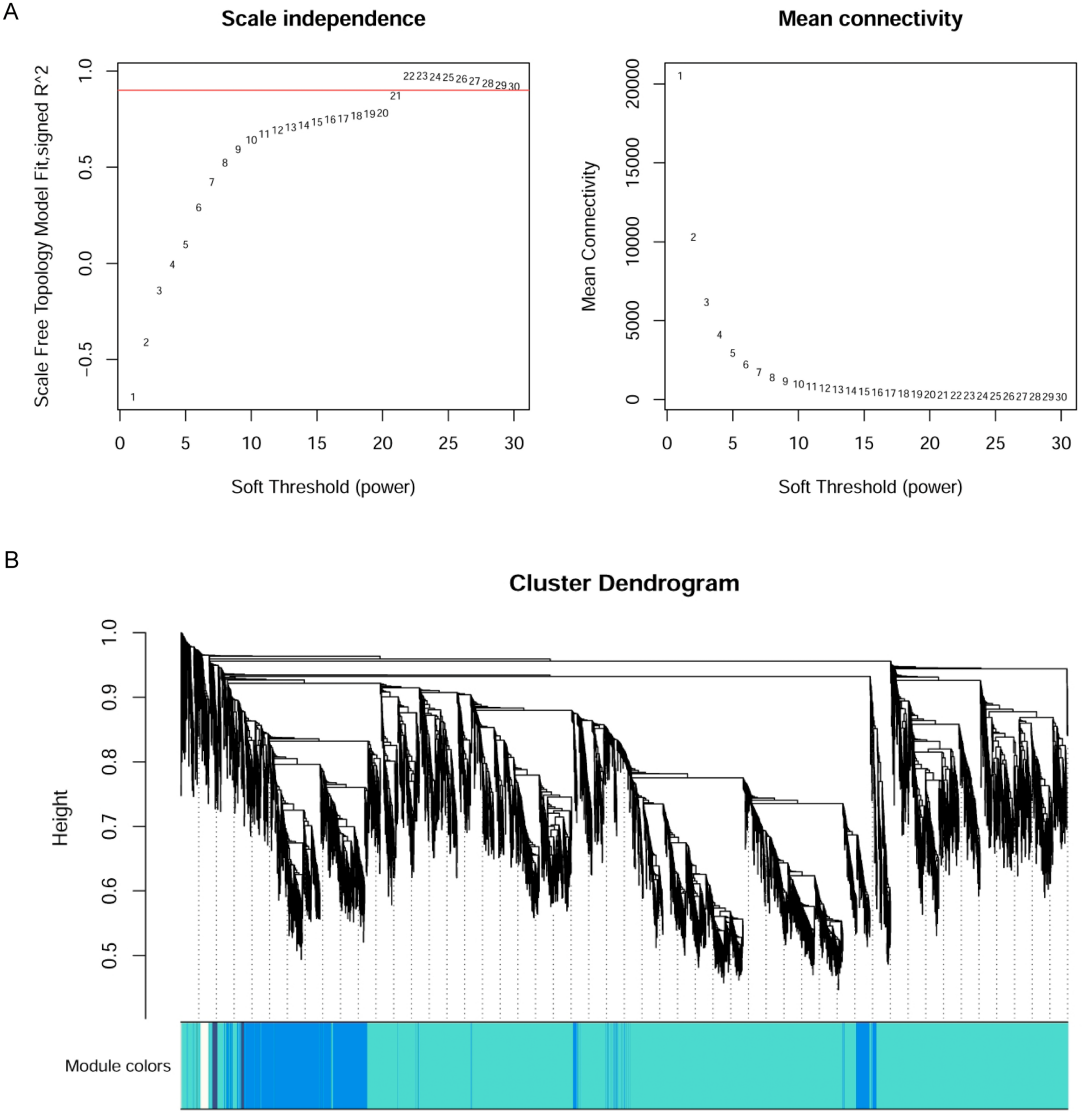
Co-expression network analysis of 11 RNA-seq datasets for wheat flower development. (A) Determination of soft threshold of WGCNA. (B) Modules identified by the dynamic tree cut and the merged modules with similar expression pattern.

**Figure 2:**
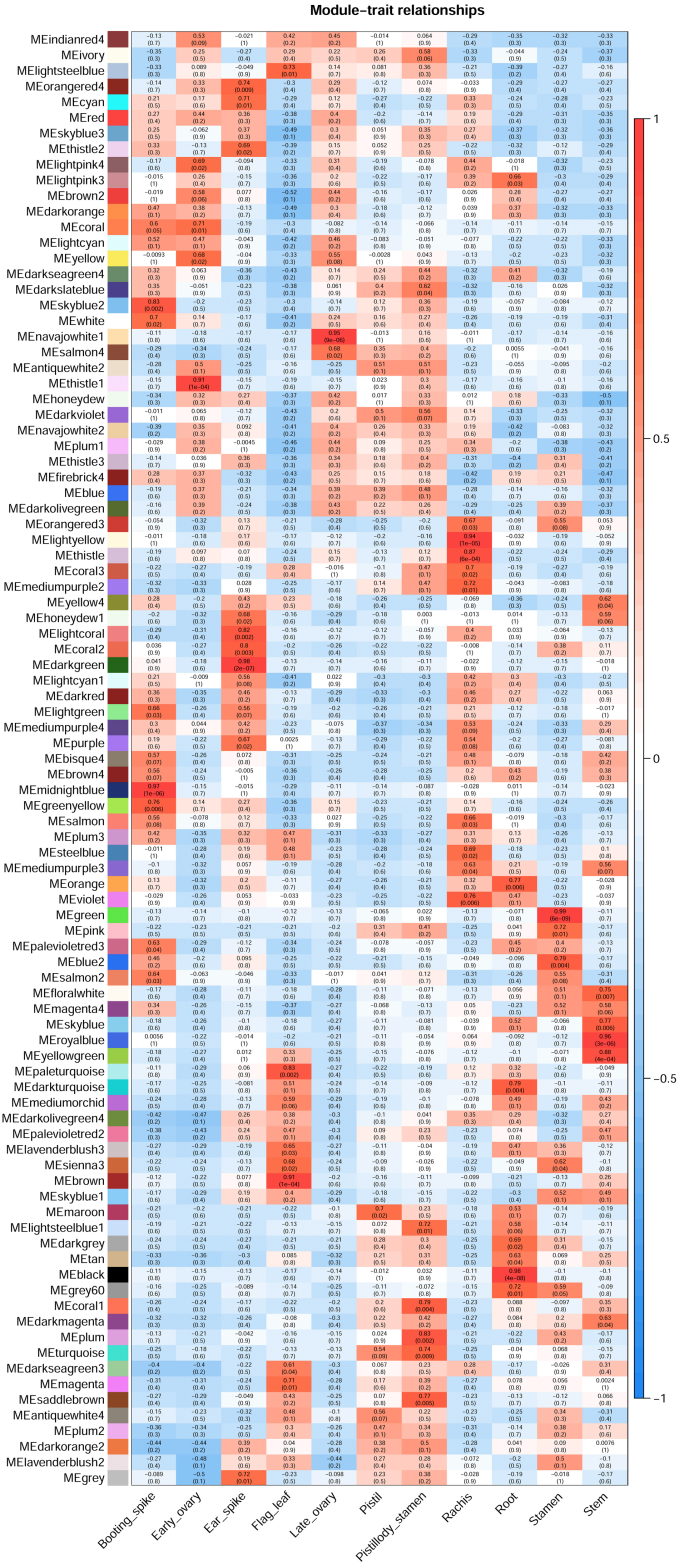
Heatmap of module–trait relationships. Each row in the heatmap represents a module. Each column represents a wheat tissue. The color of each cell represents the association coefficient between the module and the tissue type. Red color indicates the positive relationship between module and tissue type. Green color indicates the negative relationship between module and tissue type.

The WGCNA R package (Langfelder & Horvath, 2008) was applied to construct the co-expression network of the 11 wheat tissues (58,576 genes). A soft threshold (power) was set to make the genes in the network conform to the scale-free topology distribution. The first soft threshold (power) was used to construct the WGCNA using the scale-free topology-fitted index of 0.9 (Fig. 1A). Then, the block-wise Modules function was applied to construct block-wise networks with the following parameters: power = 21, networkType = “unsigned”, maxBlockSize = 10000, TOMType = “unsigned”, minModuleSize = 30, mergeCutHeight = 0.15. Figure 1B depicts the hierarchical cluster tree and merged modules. If a module was highly correlated to other modules, they should be merged into one module. The module eigengenes (MEs) were defined as the first principal component in the module, representing the gene expression profiles in this module. The module eigengenes function was applied to calculate the MEs. Then, the model matrix function was used to build a binary matrix for each trait, including 11 vectors with 11 elements each. In this matrix, when the position was equal to 1, it represented the stated trait; otherwise, the position was 0. Afterward, the correlations between MEs and traits were analyzed by module–trait relationship analysis. The *P*-values of the module–trait relationships were estimated by using the corPvalueStudent function. Then, the module–trait relationship heatmap was plotted (Fig. 2). A module was considered significantly associated with a trait, when the *P*-value of the module was less than 0.05. Afterward, the modules significantly associated with booting spike, early ovary, late ovary, ear spike, pistil, stamen, and pistillodic stamen were selected as candidate modules for further analysis.

### GO enrichment analysis of genes from candidate modules

**Figure 3:**
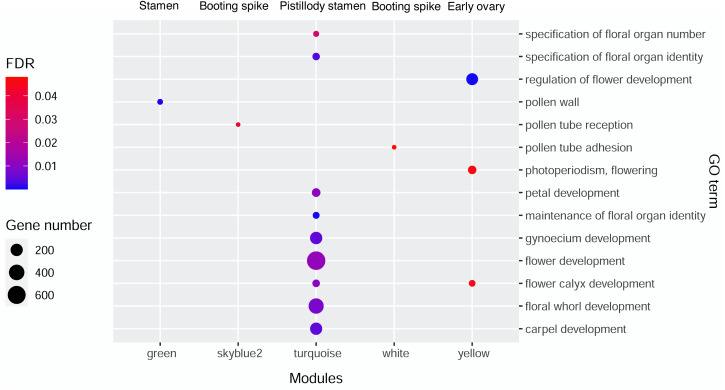
Bubble diagram of significant GO terms in wheat flower development in different modules.

AgriGO V2.0 (http://systemsbiology.cau.edu.cn/agriGOv2/index.php) (Tian et al., 2017) was employed in the GO enrichment analysis. The singular enrichment analysis (SEA) tool and the *Triticum aestivm* species were selected for this analysis. The query IDs were the Ensembl ID of wheat from the candidate modules that were remarkably related to the seven wheat flower tissues. The other parameters of AgriGO were set as follows: Fisher’s testas the statistical test method, Yekutieli (faslse discovery rate (FDR) under dependency), significance level of 0.05, one minimum number of mapping entries, and complete GO type. Table S1 displays the significant GO terms of wheat flower development from the candidate modules mentioned above with FDR values less than 0.05. Then, ggplot2 was applied to build the bubble diagram of the flower development GO terms in different modules (Fig. 3).

### Hub gene identification

Hub genes were the genes with high connectivity (top 10%) in the modules. The hub genes were determined using the absolute values of eigengene-based connectivity (kME) and gene significance (GS). kME measures the distance from the expression profile of a gene to the module eigengene, and the signedKME function in WGCNA was used to calculate kMEs. GS is the association of each gene with a trait. The hub genes from the modules that were significantly associated with pistillody were determined using the following thresholds: absolute values of GS >0.2 and kME >0.8. Then, the information of the hub genes were integrated with flower development-related genes and displayed in Table S1.

### Similarity analysis between proteins of hexaploid wheat and published pistillodic stamen proteins

The published proteins related to pistillody development are listed in Table S2, including the protein accession and origin reference information. The BLASTP program of BLAST was employed to investigate the similarity between published pistillodic stamen proteins against the obtained wheat proteins (-b 1 -m 8 -F F). A total of 172 published pistillodic stamen proteins were used as the query sequences, and the proteins of hexaploid wheat were used as the subject sequences (or database). The alignment results are shown in Table S2. The identity in Table S2 was defined as the matching base number over the number of alignment sequences. The closer the identity value to 100%, the more matched base pair between the query and subject sequences had. The coverage of query sequences was calculated using the following method to assess the similarity between the query and subject sequences. First, the alignment end position (query end) was subtracted from the start position (query start). Then, the difference was added with one. Moreover, the coverage value of thequery sequence was obtained by dividing the length of the query sequences with the sum from the previous step. In Table S2, AgriGO V2.0 was used to do the GO enrichment of the subject sequences, but only the GO terms related to wheat flower development were displayed. The subject sequences were used for further analysis, whose similarities and coverage values were up to 95% against the query sequences. Finally, the subject sequences were considered as the candidate genes of pistillody development, if they met the following criteria: (i) the GO analysis results were related to wheat carpel development (GO:0048440), or (ii) they are hub genes in significant modules.

### TF analysis

The sequences from wheat Ensembl proteins were extracted using the gene IDs of the modules that were remarkably related to pistillody to identify the TFs for pistillody development. Next, the hmmsearch program of the HMMER V3.0 package was employed to predict the TFs using proteins from the significant modules against HMM profiles. In total, 75 HMM profiles belonging to 69 TF families were used to identify the TFs (Chen et al., 2015). The default parameters of hmmsearch were employed. The proteins were considered TFs when their e-values were less than 0.01. The predicted TFs of HTS-1 are listed in Table S3. The hub gene IDs of the MEturquoise module were integrated with the predicted TFs. Then, AgriGO V2.0 was applied for the GO enrichment analysis using the same parameters above. The protein IDs of the identified TFs were used as the input data. The GO analysis results are displayed in Table S3. The published TFs related to pistillody development were surveyed (Table S4). Furthermore, the BLASTP program was utilized to compare the published TFs with the predicted TFs from HTS-1 (-b 1 -m 8 -F F) for figuring out their similarities. The BLAST results are displayed in Table S4. The TFs were considered the candidate genes regulating pistillody development if they met the following criteria: (i) the GO analysis results were related to carpel development (GO:0048440) or gynoecium development (GO:0048467); (ii) the similarity and coverage values of the BLAST alignments were less than 95%.

### Visualization of co-expression network for wheat pistillody development

**Figure 4:**
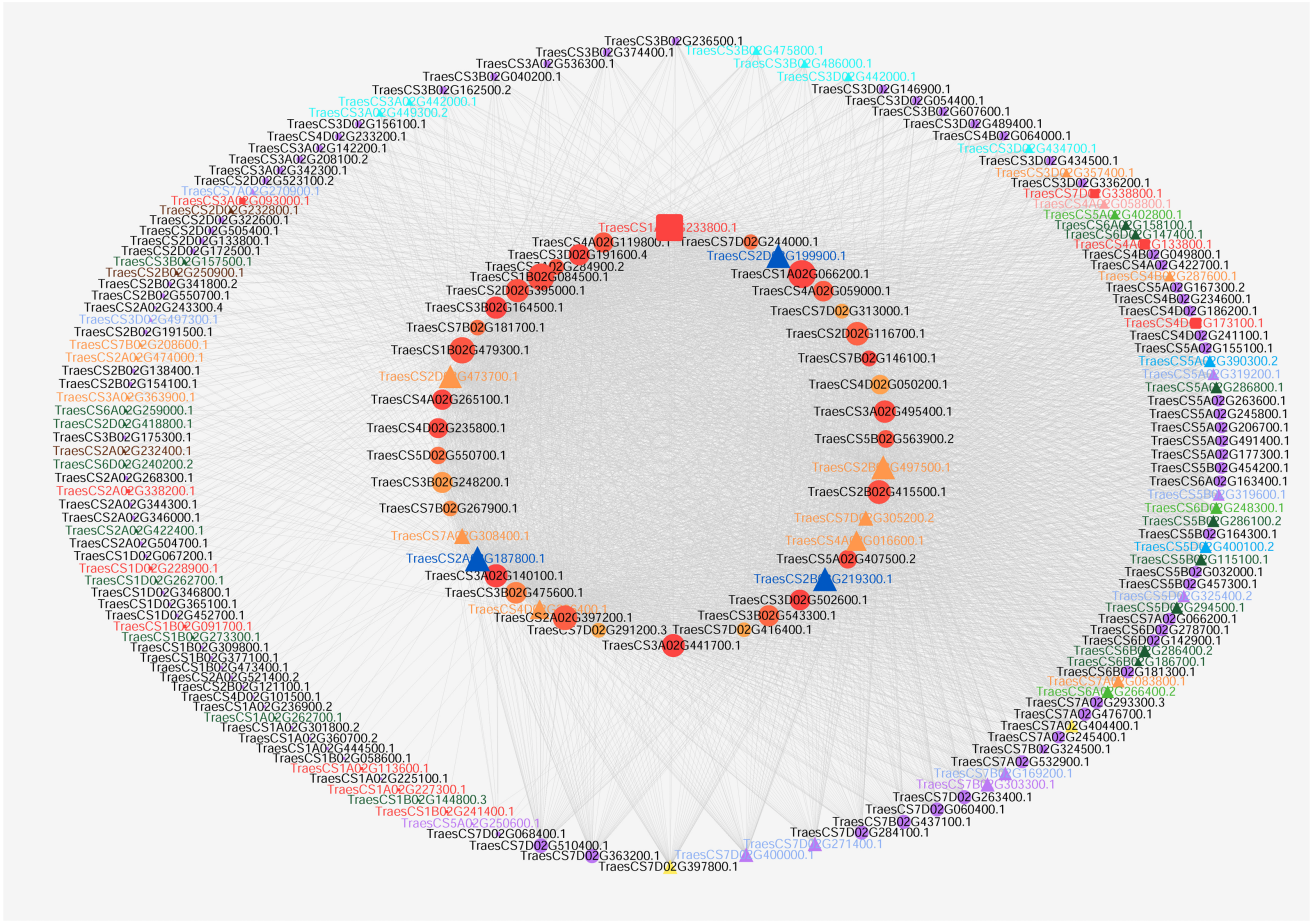
Co-expression network visualization of hub genes in the MEturquoise module associated with pistillody development. Each node represents a gene, and lines between genes represent co-expression relationships. The node size indicates the connectivity degree of the input genes. The red square nodes represent ethylene-related genes. ARF TFs are marked with turquoise triangles. BES1 TFs are marked with navy blue triangles. bHLH are marked with purple triangles. C2H2 TFs are marked with yellow triangles. GARP TFs are marked with green triangles. HB TFs are marked with orange triangles. MADS-box TFs are marked with dark green triangles. MYB TFs are marked with blue triangles. SRS TFs are marked with light purple triangles. YABBY TFs are marked with pink triangles.

According to the GO enrichment results, hub genes for carpel development (GO:0048440) and gynoecium development (GO:0048467) were obtained from Table S1. Then, flower development target nodes connected with these hub genes were identified from the pistillody-related significant correlation module. Cytoscape V3.3 was applied to visualize the networks for wheat carpel development (Shannon et al., 2003). The network only displayed the connection with a corresponding topological overlap greater than 0.08. After the data was imported, we selected Tools → NetworkAnalyzer → Network Analysis → Analyze Network. Next, general style from the statistics menu was selected to set map the node size to the degree and map the edge size to the weight. Figure 4 displayed the network of the hub genes relating to carpel development (GO:0048440) and gynoecium development (GO:0048467).

### qRT-PCR and statistical analyses

**Figure 5:**
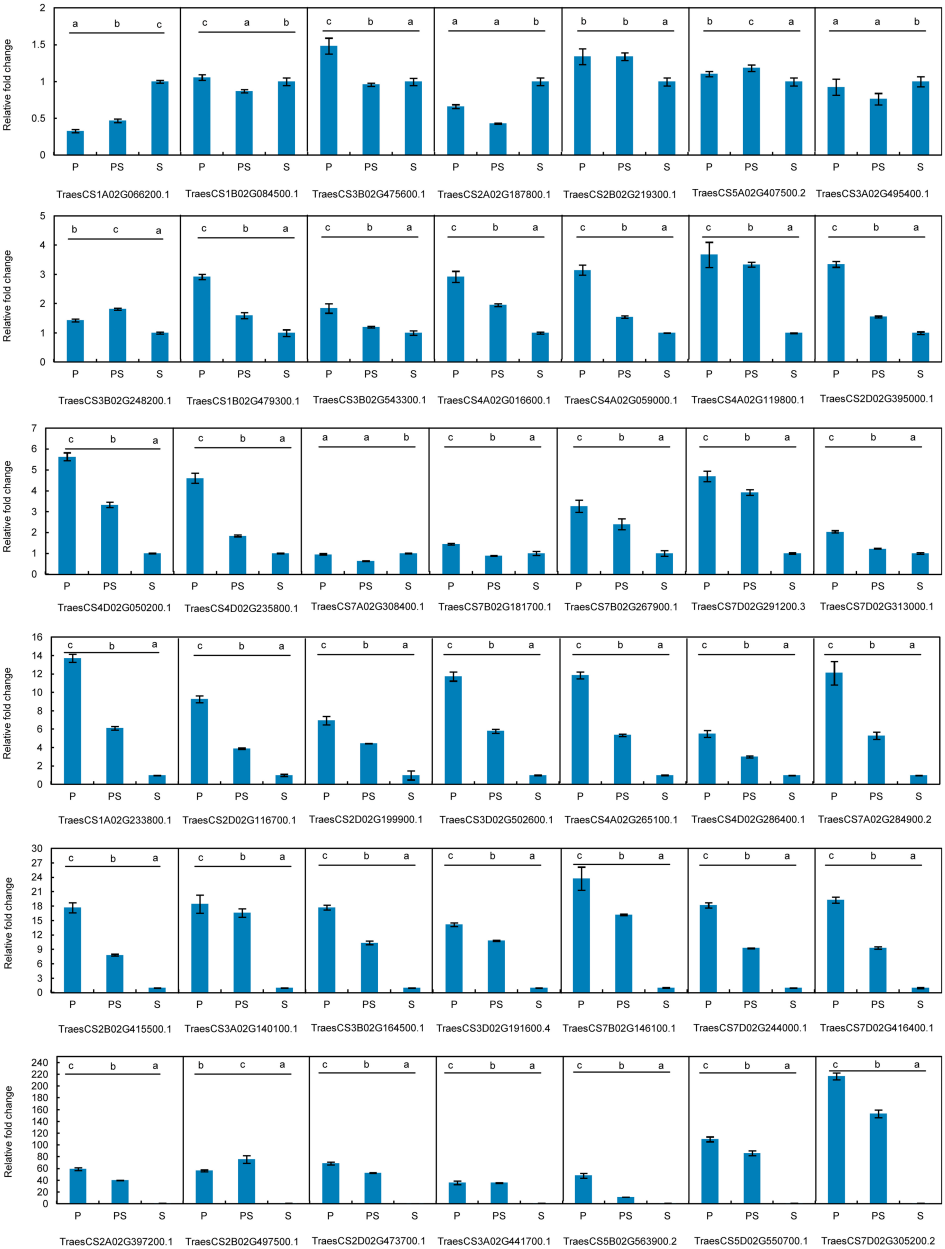
Expression patterns of 42 hub genes associated with pistillody development verified by real-time PCR. *P* represents the fully developed normal pistil of HTS-1. PS represents the completely developed wheat pistillodic stamen of HTS-1. S represents the fully developed normal stamen of CSTP.

qRT-PCR assays were performed with SsoFast™ EvaGreen (Bio-Rad, Hercules, CA, USA) using the Bio-Rad CFX96 real-time PCR platform (Bio-Rad, Hercules, CA, USA). The primers for the 42 hub genes associated with wheat pistillody development were shown in Table 1. Each biological replicate had three technical replicates. Each reaction contained 5µL of SSofast (Bio-Rad, Hercules, CA), 1 µL of gene-specific primers, 0.5 µL of cDNA and 3.5 µL of ddH_2_O, in a final volume of 10 µL. The 18S (GenBank accession number AY049040) and actin genes (GenBank accession number AB181911) were used as the reference genes (Liao et al., 2015). Fold-changes in RNA transcripts were carried out by the 2^−ΔΔCt^ method (Livak & Schmittgen, 2001). The expression patterns of the 42 genes associated with pistillody development were displayed in Fig. 5. The relative expression level of each gene in the stamen was normalized to 1.

**Table 1:** The primers of the 42 hub genes associated with wheat flower pistillody-stamen development.

Gene ID	Forward (5′-3′)	Reverse (5′-3′)	Amplifier length
18S	AAGGCGAAGATCCAGGACAAG	TGGATGTTG TAGTCCGCCAAG	107
Actin	ACGCTTCCTCATGCTATCCTTC	ATGTCTCTGACAATTTCCCGCT	121
TraesCS1A02G066200.1	ACCTCACTCCACACTCTAGCTCATC	TTCAGCGAGGAGGAAGAGTAGGATG	134
TraesCS1A02G233800.1	CACTATGGCGACTCGGCATGTTC	TCAAGAGAGACGACACCGGGATG	115
TraesCS1B02G084500.1	CCCTCCTCCTCCTCAGCATCATC	AGGCGGATGGTGTTGTTGTTGAG	145
TraesCS1B02G479300.1	GCGGAGATCAAGGTGCTCAACTAC	GTCAGGCTCTTCATCCCGCTAATC	92
TraesCS2A02G187800.1	GGAAGGAGCGGGAGAACAACAAG	AGTGCTTGGGCAGCTTGTAGTTG	108
TraesCS2A02G397200.1	CAGGCGGCTCAGAGAGGTCTATC	GGTTCTTCGGTACTGACAGTGTTTCC	107
TraesCS2B02G219300.1	GACACGATACCGGAGTGCGA	ATGGAGTTGGAGCTGGAGGC	140
TraesCS2B02G415500.1	AGGTGCTTGCTCTTGAGGACAAC	AGTTCCCACCAAGATAGACCTCTCTG	96
TraesCS2B02G497500.1	GCATCGTCCAGGAAATGGCTCAC	GTTGGTGCTATTGACACGGAGGAG	83
TraesCS2D02G116700.1	CCATTGCCGTCGCCATACTCATC	TCCTCCTCCTCGAACTCCACATTG	148
TraesCS2D02G199900.1	GACACGATACCGGAGTGCGA	ATGGAGTTCGAGCTGGAGGC	140
TraesCS2D02G395000.1	ACGAAGCTGGTCATGTTCAACTCC	CCACGGCTAAGCACATTCTCCTC	92
TraesCS2D02G473700.1	TCGTCCAAGAAATGGCTCACATCG	GTTGGTGCTATTGACACGGAGGAG	80
TraesCS3A02G140100.1	AAGGCTACACACCGGACCTCTTG	GGTGGTGGTGGTGGTGAAACATAG	105
TraesCS3A02G441700.1	GAATGTGATGCTGGAAGAAGGGTTTG	CACTTATCCGTGTACCTGCTACTTTGG	150
TraesCS3A02G495400.1	ACATCGGGAAGTGCTCCTCTCTC	GTATCTCGCCGGTGAGGTTGTTG	138
TraesCS3B02G164500.1	GCCAGAGCCAGTTCTTCTTCCAG	ATCGGGTTGTTGTCGTCAGCTTC	103
TraesCS3B02G248200.1	CGTCCTCTCCCACAACAATCTGTC	ATGCTGGGGATTTTGCCTTCGAG	114
TraesCS3B02G475600.1	TCTACACCGCTCCAGAGTGCTAC	AAATGGGTCCGAGGGGTCTTTCC	116
TraesCS3B02G543300.1	AGAGAAGATCCTCGGAAGGCTGTAC	GTGTCGTAAGGCAGGTCGTTCAG	102
TraesCS3D02G191600.4	ATGCTGGAATGATTGGGACTACAAGG	ATATGGCTGTCGTGCTCCTTTGATAC	135
TraesCS3D02G502600.1	GAAGTGCTCCTCCCTCTACCTACTG	GAGGTTGTTGTTCTCCAGCCGTAG	118
TraesCS4A02G016600.1	ATCGACCATCTCCTCCACGC	AGAGGAGGTTGCCGAAGCTG	85
TraesCS4A02G059000.1	CGACCAAGGAAGGCGTGATGAAG	CAGAGCATGGCGACGTAGAAGAC	94
TraesCS4A02G119800.1	GAGGATATAAGAAGGTGGATGGGCAAC	AGGATTTCAAGTGTTTGTCTGGAGGAG	110
TraesCS4A02G265100.1	CTGAGTTGCCAGCACTGAGAAAGG	TGCCATCAAGTATCACCTCCACATTC	99
TraesCS4D02G050200.1	TGAAGCGAATGGAGTCTGGTGTTATG	AAGCAGCGATACAAGGTTCCTATGAC	107
TraesCS4D02G235800.1	CCAAGGAAGGCGTGACGAAGATC	CAGAGCATGGCGACGTAGAAGAC	91
TraesCS4D02G286400.1	ATCGACCATCTCCTCCACGC	ACAGCAGGTTGCCGAAGCT	85
TraesCS5A02G407500.2	GAATCCAACACTCCTGCTCCAACC	TGCTGCTGCTGCTGATGATGATG	83
TraesCS5B02G563900.2	TGCTGCCATATCTGCTGGAAACG	ACGATGATGATTCAATGGAGAGGACAC	104
TraesCS5D02G550700.1	GCTGCTTTGATTCGTGAAGTTGCTAG	GCCCTTCTTAATTGACACTGGATTTGC	147
TraesCS7A02G284900.2	CCGAGATTTCCTGCTGGATGTGG	TCGGCGTTGATGGCATAGATGAC	145
TraesCS7A02G308400.1	CATCTTCTTCTTCCCCAGCACCAC	TCATCTCCAGCTCTTCGTTGTTGTTG	127
TraesCS7B02G146100.1	CAGACGAGAAGCTGCTCATCTACG	CCACGGTAATGGAGTTGGCGAAG	98
TraesCS7B02G181700.1	TCGTCGTCAGGTACGCCTTCTAC	CGGTCCACATCCAGCAGGAAATC	90
TraesCS7B02G267900.1	GCCTCCAAATGCTCAACCTTTCTTAC	CTAGGAACGATTCTGCGGTGAAGC	93
TraesCS7D02G244000.1	AGAGAGTGAGGAGCCAAGAATCCC	ACCACAATGACGGCAAGAATCGG	126
TraesCS7D02G291200.3	GATGTCGGAAGTGGTAAGGATGCTC	CGTGGAGGTTGTCAGTGGAATCG	149
TraesCS7D02G305200.2	ACAACAACGAAGAGCTGGAGATGAG	TGACGGTGGTAACGCTTCTTCTTC	145
TraesCS7D02G313000.1	AACACTGGACCTCCGCAACAATAC	CGTTAGGACAGGCTTTCAGAGAACC	137
TraesCS7D02G416400.1	TTCGCCGTGTCCTGTCTTCTTTG	GCCCTCCAAATCTCTACCACCTTG	87

The differences in the relative gene expression levels among the pistil, pistillodic stamen, and stamen were determined by one-way ANOVA followed by least significant difference test. Statistical analyses were performed by SPSS V26 software at the significance level of *p* < 0.05.

## Results

### Overview of WGCNA networks construction and module detection

The 58,576 genes obtained by Salmon were used to construct WGCNA networks for investigating the molecular mechanism of pistillodic stamens in HTS-1. Figure 1A showed that the soft threshold (power) of 21 was used to construct the networks. Figure 1B showed the gene dendrogram clustering on Tom-based dissimilarity and modules obtained by the dynamic tree cut. Figure 1B depicted the major tree branches that constitute 93 merged modules labeled by different colors (cutHeight = 0.15). The gene number in the modules ranged from 32 to 8,764 with an average of 630 genes per module.

### Identification of significant modules related to trait

Figure 2 revealed the relationships between the modules and tissue types. A module has a significant correlation with the tissue type when *p* < 0.05. Figure 2 depicted that the number of significant modules associated booting spike, early ovary, ear spike, late ovary, pistil, pistillodic stamen, and stamen development were 7, 4, 8, 2, 1, 6, and 4, respectively. Figure 2 displayed that MEsalmon2, MEpalevioletred3, MEgreenyellow, MEmidlightblue, MElightgreen, MEwhite, and MEskyblue2 were remarkably related to booting spike development. The MEthistle1, MEyellow, MEcoral, and MElightpink4 modules were the modules remarkably associated with early ovary development. The modules considerably related to pistillodic stamen development were MEturquoise, MEsaddlebrown, MEplum, MEcoral1, MElightsteelblue1, and MEdarkslateblue. MEsienna3, MEblue2, MEpink, and MEgreen were remarkably related to stamen development (Fig. 2).

### Gene Ontology (GO) enrichment analysis of genes from significant modules

Gene Ontology enrichment analysis by AgriGO V2.0 was performed to investigate the genes related to wheat floral organ development in the significant modules displaying in Fig. 2. Table S1 showed the genes of significant GO terms about wheat floral organ development identifyied by AgriGO, such as stamen, early ovary, and pistillody stamen development. In Table S1, 1,844 genes were substantially related to wheat flower development. A bubble diagram was employed to clearly illustrate the flower development-related genes (Table S1), whose FDR values were less than 0.05. In Fig. 3, the MEgreen module enriched eight genes about pollen wall, and the MEgreen module was remarkable related to stamen development (Fig. 2). The MEskyblue2 module identified three genes about pollen tube reception, and three pollen tube adhesion genes were found in the MEwhite module (Table S1). The MEskyblue2 and MEwhite modules were remarkably associated with booting spike development (Fig. 2). In the MEyellow module, remarkably relating to early ovary development in wheat, 180, 18, and 53 genes participated in regulating flower development, flower calyx development, and flowering photoperiodism, respectively (Table S1). The MEturquoise module, which was remarkably related to pistillodic stamen development, enriched genes related to the specification of floral organ number (10 genes), the specification of floral organ identity (31 genes), the maintenance of floral organ identity (21 genes), flower development (645 genes), flower calyx development (33 genes), floral whorl development (377 genes), carpel development (198 genes), gynoecium development (206 genes), and petal development (58 genes). Next, the genes related to carpel and gynoecium development were investigated. The analysis implied that 198 genes for carpel development can be found in the 206 genes for gynoecium development. Moreover, Table S1 also showed the hub gene information of 1,844 genes in the MEgreen, MEskyblue2, MEturquoise, MEwhite, and MEyellow modules. None of the hub genes was found in the other four modules, except in the MEturquoise module. A total of 264 hub genes were found in the MEturquoise module (accounting for 14.3% of the 1,844 genes). Among the 264 hub genes, 42 hub genes were related to carpel (GO:0048440) and gynoecium development (GO:0048467). The IDs of the 42 hub genes were listed in Table 1.

### Similarity analysis between proteins of HTS-1 and the published pistillody development proteins

The BLASTP program was used to compare the similarity between the published pistillody development proteins and the proteins of hexaploid wheat (Table S2). In Table S2, the alignment results included the identity, start and end positions, expect value, and score. In the eleventh column, we calculated the coverage parameter of query sequences. If the coverage and identity of query sequence were 100% similar, then the query sequence was the same as the subject sequence. 24 subject sequences had 100% coverage and identity against the query sequences. The expect values of the 24 proteins were very close to zero. None of the 24 subject sequences was found in the 42 hub genes listed in Table 1. A total of 80 query sequences were highly similar with the subject sequences, whose identities and coverage values between them were up to 95%, respectively (Table S2). Among the 80 subject sequences, only *TraesCS2A02G397200.1* was hub genes in MEturquoise module (Table 1). The 12th to the 17th columns of Table S2 provided the GO annotation information of the subject sequences related to wheat flower development. Three GO terms about pistil development were found. The number of sequences related to plant ovule development (GO:0048481), plant type ovary development (GO:0035670), and carpel development (GO:0048440) were 7, 7, and 10, respectively (Table S2). Thus the number of sequences about wheat pistil development was 10 (Marked with green background in Table S2). According to the GO analysis, *TraesCS2A02G397200.1* and *TraesCS3D02G502600.1*, which were associated with anther and carpel development, were found in the 42 hub genes listed in Table 1. Furthermore, the significant module information of the subject IDs were provided in the 18th column of Table S2.

### Identification of TFs about pistillody development

The TFs in the MEturquoise module were listed in Table S3. A total of 618 TFs were identified from 50 different families in the MEturquoise module (Table S3). Then, the TFs were integrated with the hub gene information of the MEturquoise module listed in Table S1. In total, 62 of the 618 TFs were hub genes (accounting for 10%, Table S3). Moreover, AgriGO V2.0 was used in the GO enrichment of the 618 TFs. 17 GO terms, including GO:0048440 (carpel development), GO:0048467 (gynoecium development), and GO:0048443 (stamen development), were associated with wheat flower development. Among them, 67 TFs were related to carpel development (GO:0048440) or gynoecium development (GO:0048467). The 67 TFs were identified from 12 different families. The number of TFs from different families were as follows: auxin response factors (ARFs, 6 genes), brassinosteroid insensitive 1-EMS-suppressor 1 (BES1, 3 genes), basic helix-loop-helix (bHLH,2 genes), Cys2His2 (C2H2, 2 genes), GARP (Golden2, ARR-B, Psr1; 3 genes), homeobox (HB, 13 genes), MIKC of MADS-box (13 genes), M-type of MADS-box (6 genes), MYB (7 genes), squamosa-promoter binding protein (SBP, 3 genes), SHI related sequence (SRS, 8 genes), and YABBY (1 gene). Among the 67 TFs, 9 hub genes were from the BES1 (3 genes) and HB families (6 genes). Subsequently, the similarities of the published wheat TFs for pistillody development were aligned against the 618 TFs by BLASTp (Table S4). 25 published TFs had more than 95% of identity and coverage values with 15 TFs in the MEturquoise module, whose families were MADS-box, YABBY, and MYB families (Table S4). 20 of the 25 published TFs had high similarity with 12 different MADs-box genes identified in the MEturquoise module. 4 of the 25 published TFs were highly similar to 2 YABBY sequences. The remaining one was identified in the MYB family. Among the 25 TFs from the MEturquoise module, GO analysis showed that 18 TFs participated in regulating carpel development (GO:0048440). In particular, two published MADS-box TFs (*ALM58843.1* and *TraesCS6A02G259000.1*) had 100% of identity and coverage values with the TFs in the 618 sequences (Table S4).

### Visualization of co-expression network about pistillody development

Cytoscape V3.3 was utilized to visualize gene networks for wheat pistillody development in the MEturquoise module. Figure 4 depicted the co-expression network relating to wheat carpel development (GO:0048440) and gynoecium development (GO:0048467). 42 hub genes for wheat pistil development were located in the inner circle, and 142 nodes connected with the 42 hub genes were located in the outer circle (Fig. 4). The node size represented the connectivity degree of the nodes. The bigger the node, the more connectivity that node had in the networks. The top 10 nodes among the 42 nodes by connectivity were: *TraesCS1A02G066200.1*, *TraesCS1A02G233800.1*, *TraesCS1B02G084500.1*, *TraesCS1B02G479300.1*, *TraesCS2A02G187800.1*, *TraesCS2A02G397200.1*, *TraesCS2D02G199900.1*, *TraesCS2B02G219300.1*, *TraesCS2B02G497500.1*, and *TraesCS2B02G415500.1*. In total, 11 ethylene-related genes were identified using AgriGO, and the ethylene-related genes were marked with red squares and labeled in red (Fig. 4). Among the 11 ethylene-related genes, only TraesCS4A02G133800.1 is one of the top 10 hub genes. Among the 42 hub genes, three BES1 TFs (marked with navy blue triangle) were connected with five ethylene-related nodes. The IDs of the BES1 TFs are *TraesCS2A02G187800.1*, *TraesCS2B02G219300.1*, and *TraesCS2D02G199900.1*. The ethylene-related nodes are *TraesCS2A02G338200.1*, *TraesCS4A02G133800.1*, *TraesCS4D02G173100.1*, *TraesCS3A02G093000.1*, and *TraesCS7D02G338800.1*. Five of the HB family hub genes were linked to four of the ethylene-related genes in the networks. The HB hub genes are *TraesCS2B02G497500.1*, *TraesCS2D02G473700.1*, *TraesCS4A02G016600.1*, *TraesCS7D02G305200.2*, and *TraesCS7A02G308400.1*, and the ethylene-related genes connected with them are *TraesCS3A02G093000.1*, *TraesCS4A02G133800.1*, *TraesCS4D02G173100.1*, and *TraesCS7D02G338800.1*. Furthermore, the TFs of the 142 nodes in the outer circle were analyzed. 52 TFs belonging to 11 families were identified. Among them, 17 MADS-box and 13 HB TFs were identified, which accounting for 32.7% and 25% of 52 TFs, respectively. GO analysis by AgriGO revealed that only 2 TFs were associated with ethylene: *TraesCS1B02G091700.1* (HB family) and *TraesCS2A02G338200.1* (MYB family).

### qRT-PCR verification of hub genes about pistillody development

The expression profiles of the 42 hub genes listed in Table 1 were validated by qRT-PCR to validate the genes that regulate wheat pistillody development (Fig. 5). In Fig. 5, the expression features were divided into three categories. The first category was that the relative expression levels of pistil and pistillodic stamen were lower than that in the stamen. This category maintained four genes: *TraesCS1A02G066200.1*, *TraesCS2A02G187800.1*, *TraesCS3A02G495400.1*, and *TraesCS7A02G308400.1*. The second category was that the relative expression levels of genes among the three flower tissues were less than two times, and the gene expression levels in the pistillodic stamen or pistil were slightly higher than those in the stamen. The genes in the second category were: *TraesCS1B02G084500.1*, *TraesCS3B02G475600.1*, *TraesCS2B02G219300.1*, *TraesCS5A02G407500.2*, *TraesCS3B02G248200.1*, *TraesCS3B02G543300.1*, and *TraesCS7B02G181700.1*. The last category was composed of the remaining 31 genes. In this category, the gene expression levels in the pistillodic stamen and pistil were remarkably higher than those in the stamen. The expression levels of *TraesCS7D02G305200.2* in the pistillodic stamen and pistil were more than 150 fold greater than that in the stamen. The results of ANOVA showed that the expression levels of four genes in the pistil and pistillodic stamen were remarkably different from those in the stamen. The IDs of the four genes were: *TraesCS2A02G187800.1*, *TraesCS1B02G479300.1*, *TraesCS3A02G495400.1*, and *TraesCS7A02G308400.1*. In addition, the expression levels of the remaining 38 genes in the pistil, pistillodic stamen, and stamen were significantly different (*p* < 0.05).

## Discussion

Pistillody development is a rare phenomenon of male sterility in common wheat (Murai et al., 2002; Yamamoto et al., 2013; Peng et al., 2013). However systematical identification of pistillody-related genes and their interaction networks have never been reported. In this research, WGCNA was applied to identify hub genes in tissue-specific modules and explore gene interaction networks from RNA-seq of 11 wheat tissues. Six modules were remarkably associated with the development of the pistillodic stamen of HTS-1, such as MEturquoise, MEsaddlebrown, and MEplum (Fig. 2). However, the 206 genes correlated to wheat carpel or gynoecium development were identified only in the MEturquoise module (Table S1). Meanwhile, correlation values of the MEturquoise module about pistillodic stamen, pistil, and stamen were 0.74, 0.54, and 0.068, and their *p* value were 0.009, 0.09, and 0.8 (Fig. 2), respectively. This result indicated that genes of the MEturquoise module were positively correlated with pistillodic stamen development. In addition, eight genes about pollen wall in the MEgreen module were enriched in the stamen. The correlation values of the MEgreen module about pistillodic stamen, pistil, and stamen were 0.022, −0.065, and 0.99, respectively (Fig. 2). It demonstrated that the MEgreen module had highly positive correlation with stamen development. Pollen tube reception and pollen tube adhesion genes were enriched in the MEwhite and MEskyblue2 modules of booting spike. Some genes about flower development (180 genes), flower calyx development (18 genes), and flowering photoperiodism (53 genes) were remarkably associated with early ovary development (Table S1, Fig. 3). These results indicate that the module classification of WGCNA is accurate. The gene expression patterns of the 42 hub genes were validated by qRT-PCR to verify whether the candidate genes were related to pistillody development (Fig. 5). Figure 5 displayed that most of the 42 genes, except four genes, had higher expression levels in the pistil and pistillodic stamen comparing with those in the stamen. The IDs of the four genes were: *TraesCS1A02G066200.1*, *TraesCS2A02G187800.1*, *TraesCS3A02G495400.1*, and *TraesCS7A02G308400.1*. The q-PCR results imply that the 38 hub genes associated with pistillody development identified by WGCNA are very reliable (Fig. 5); and the 38 hub genes play critical roles in pistillody development of HTS-1.

The development of flower features directly affects crop yield and other agronomic traits. Male sterility has been widely studied in flower development and has very important application value in breeding (Peng et al., 2013). Stamen transformation into pistil or pistil-like structure (called pistillodic stamen) is a rare phenomenon in male sterility. Previous studies reported some pistillodic stamen material in wheat, such as (cr)-n26, (cr)-csdt7bs, and HTS-1 (Murai & Tsunewaki, 1993; Murai et al., 2002; Peng et al., 2013), which provided some insight into understanding the molecular interaction mechanism of wheat pistillody genes (Murai et al., 2002; Zhu et al., 2008; Yamamoto et al., 2013; Yang et al., 2015). The published pistillody-related proteins were compared with the proteins of HTS-1 by BLAST. The alignment results have been shown in Table S2. The BLAST results display that the protein of the *WAP3* gene (NCBI accession number *BAA33459.1*) (Murai et al., 2002) is highly similar with the hexaploid wheat proteins of *TraesCS7B02G286600.2* (highlighted with a green background in Table S2). The identity value and the ratio of alignment sequence that cover the total length between the *WAP3* gene and the HTS-1 genes were greater than 99% (Table S2). Murai et al. (2002) reported that the decreased expression level of *WAP3* could induce the pistillody of (cr)-csdt7bs. *TraesCS7B02G286600.2* was confirmed to be related to stamen development (GO:0048443) and carpel development (GO:0048440, Table S2). Therefore, this result consists with the published results of Murai et al. The protein of the mitochondrial gene *orf25* (*BAA82046.1*) is a candidate gene related to pistillody in alloplasmic wheat (Hama et al., 2004). *BAA82046.1* had 100% of similarity and coverage with *TraesCS4D02G236200.1* in HTS-1 (highlighted with a red background in Table S2). This result suggests that *TraesCS4D02G236200.1* can induce the pistillody of HTS-1. The protein of the *orf260*^*cra*^ gene (*BAG84632.1*) is associated with pistillody induction (Zhu et al., 2008). *TraesCS7A02G237800.1* in HTS-1 had 94% identity and 52% coverage with *BAG84632.1* (highlighted with a red background in Table S2). This result implies that *TraesCS7A02G237800.1* might be associated with pistillody development and can be used as acandidate gene for further study. The protein of the *WCBP1* gene (BAM65843.1) is remarkably up regulated in the young spikes of pistillodic stamens in (cr)-csdt7bs inducing by mitochondrial retrograde signaling (Yamamoto et al., 2013). *BAM65843.1* shows high similarity with the HTS-1 proteins of *TraesCS5D02G102100.1*, whose identity and coverage ratio are greater than 97% (highlighted with a red background in Table S2). Thus, *TraesCS5D02G102100.1* could be associated with pistillody development. The *WPPK1* gene (*BAF79635.1*) is strongly expressed in developing pistils and pistil-like stamens (Saraike et al., 2007). *BAF79635.1* had a high similarity with *TraesCS2B02G309900.1*, which identity and coverage were up to 99% (highlighted with a red background in Table S2). The result suggests that *TraesCS2B02G309900.1* could be associated with pisillody development. Yang et al. (2015) reported the 167 proteins of 206 DEG that might be related to the flower development of HTS-1. 80 of the 167 protein sequences were highly similar to 107 of the subject sequences, whose identities and coverage values were above 95% (Table S2). 4 proteins of the 107 proteins were associated with carpel development (GO:0048440) (marked with a green background in Table S2). In addition, there were 10 genes about carpel development (marked with a green background in Table S2). These results indicate that the 10 proteins have important roles in the development of pistillody in HTS-1. Sun et al. (2019) reported that the *TaEPFL1* gene (comp109492_c0) played an important role in stamen development and *TaEPFL1* over expression results in pistillodic stamens. The alignment identity and coverage of the *TaEPFL1* gene against *TraesCS6D02G296500.1* were 100% and 75%, respectively (highlighted with a red background in Table S2). Therefore, *TraesCS6D02G296500.1* is considered to be a candidate gene related to the development of wheat pistillody. Finally, 15 pistillody development associated genes were validated by BLAST in HTS-1 (shown with a red or green background in Table S2), which will be considered candidate genes related to pistillody development for further studies. In particular, *TraesCS2A02G397200.1* and *TraesCS3D02G502600.1* are belonging to the 42 hub genes (Table 1). This result demonstrates that *TraesCS2A02G397200.1* and *TraesCS3D02G502600.1* have important roles in pistillody development.

TFs play an important role in regulating plant development. Some previous studies reported that MADS-box (Hama et al., 2004; Meguro et al., 2003; Zhang et al., 2015; Wang et al., 2015; Yang et al., 2017; Su et al., 2019; Paolacci et al., 2007) and the *DL* gene of the YABBY family (Murai, 2013; Ishikawa et al., 2009; Zhang et al., 2015) were associated with wheat pistillody development. The results provide insights into the effect of other TF families on the mechanisms of the regulation of pistillody development. 67 TFs regulating carpel development (GO:0048440) or gynoecium development (GO:0048467) were identified from the MEturquoise module (Table S3). 19 of MADS-box TFs (MIKC, 13 genes; M-type, 6 genes) were accounting for 28.36% of the 67 sequences (Table S3). This finding suggests that the MADS-box gene plays an important role in the regulation of pistillody development. In addition, 12 TF families were found to regulate carpel development (Table S3). Among the 67 TFs, the HB, MYB, and SRS families comprised 13, 7, and 8 sequences, respectively (Table S3). The results indicate that the mechanism of pistillody development is very complex, and many TFs from different families work together to regulate the pistillody development. In addition, 9 hub genes from the BES1 and HB families were identified in the 67 TFs (Table S3). The BLASTP program was used to determine the similarity of the published TFs with the 618 TFs in the MEturquoise module. Twenty-five published TF sequences had more than 95% similarity and coverage with the 15 TFs in the MEturquoise module (Table S4). Thus, the 15 different TFs need to be removed from the 67 sequences. The 15 TFs were highlighted with a red background in Table S3 . Finally, 56 TFs from 11 families remained (highlighted with green background in Table S3).These TFs provide useful information for further research on the mechanism of pistillody development.

As a natural mutant of male sterility, HTS-1 has great value in scientific research and genetic breeding. Figure 4 displays the networks of genes associated with carpel development in HTS-1. 3 BES1 and 6 HB TFs associated with pistillody development were identified in the 42 hub genes (Table S3), and 11 ethylene-related genes were identified from all nodes (Fig. 4). Previous studies reported that the *BES1* gene played an important role in plant reproduction, including flowering and the development of ovules, pollen, and seeds (Li et al., 2018). Low BES1 levels can inhibit ethylene biosynthesis (Li et al., 2018). Moreover, ethylene plays a remarkable role in sex determination, and ethylene-related genes may result in themale and female differentiation of flowers (Sun et al., 2019; Wang, Kong & Hong, 2009; Du et al., 2016). 3 BES1 hub TFs were identified in HTS-1 (marked by navy blue triangles in Fig. 4). 2 of 3 BES1 hub genes (*TraesCS2B02G219300.1* and *TraesCS2D02G199900.1*) had higher expression levels in the pistil and pistillodic stamen than in the stamen (Fig. 5). This result indicates that *TraesCS2B02G219300.1* and *TraesCS2D02G199900.1* may positively regulate pistillody in HTS-1. The higher expression levels of *BES1* genes in HTS-1 can increase ethylene, which can result in the transformation of stamens to pistils or pistil-like structures. Previous studies reported that HB TFs determined the number of flower organ and the flowering time in wheat (Li et al., 2020). However, in *Arabidopsis thaliana*, the *HB* gene of KNOTTED-like from *A. thaliana* 2 (KNAT2) played a role in carpel development. All ovules of a KNAT2-GR lfy-6 plant were converted to pistils (Pautot et al., 2001). In total, six hub genes of HB TFs were identified among the 42 hub genes for carpel development (marked in orange triangles in Fig. 4). The qPCR results exhibited that the expression levels of the six HB genes in the pistil and pistillodic stamen were greater than those in the stamen (Fig. 5). These results suggest that HB TFs may positively regulate pistillodic development in HTS-1. A total of 17 MADS-box and 13 HB TFs were identified from 142 nodes (marked in dark green and orange triangles in Fig. 4, respectively). These results illustrate that MADS-Box and HB TFs play an important role in regulating pistil and stamen differentiation in plants. Moreover, *TraesCS1B02G091700.1* (HB family) and *TraesCS2A02G338200.1* (MYB family) are the ethylene-related genes in the networks (Fig. 4). Four hub genes, namely, *TraesCS1A02G066200.1*, *TraesCS1A02G233800.1*, *TraesCS1B02G084500.1*, and *TraesCS1B02G479300.1* were linked to the ethylene-related genes (Fig. 4). In particular, *TraesCS1A02G233800.1* is an ethylene-related gene linked to another ethylene-regulating gene (*TraesCS1B02G0917001*, Fig. 4). This result indicates that these genes play an important role in pistillody development by regulating ethylene-related genes.

## Conclusions

The current study identified 38 hub genes from the MEturquoise module, which played important roles in the pistillody development of HTS-1. A total of 15 pistillody-associated proteins were validated in HTS-1 by BLAST. 56 TFs were considered to regulate the development of pistillodic stamen. The co-expression networks illustrated that ethylene-related genes played an important role in pistillody development. The results provide insights into the psitillody development of HTS-1.

##  Supplemental Information

10.7717/peerj.13902/supp-1 Table S1 Genes significantly associated with the wheat flower development of HTS-1 identified by GO annotation from candidate modules

10.7717/peerj.13902/supp-2 Table S2 BLAST results between the published pistillody-associated proteins and HTS-1 proteins

10.7717/peerj.13902/supp-3 Table S3 Predicted transcription factors of HTS-1 identified in the MEturquoise module significantly related to pistillodydevelopment in HTS-1

10.7717/peerj.13902/supp-4 Table S4 Similarity of published transcription factors with the transcription factors identified in HTS-1 by BLAST

## Additional Information and Declarations

### Data Availability

The following information was supplied regarding data availability: The RNA-seq datasets are available at the NCBI SRA database: ERR424732, ERR424762, SRR3068439, SRR6802610, SRR6802611, SRR6802608, SRR6802613, SRR6802612, SRR1175868, SRR1177760, and SRR1177760.

## References

[ref-1] Chen ZY, Guo XJ, Chen ZX, Chen WY, Liu DC, Zheng YL, Liu YX, Wei YM, Wang JR (2015). Genome-wide characterization of developmental stage- and tissue-specific transcription factors in wheat. BMC Genomics.

[ref-2] Du SH, Sang YL, Liu XJ, Xing SY, Li JH, Tang HX, Sun LM (2016). Transcriptome profile analysis from different sex types of *Ginkgo biloba* L. Frontier in Plant Science.

[ref-3] Greenside P, Shimko T, Fordyce P, Kundaje A (2018). Discovering epistatic feature interactions from neural network models of regulatory DNA sequences. Bioinformatics.

[ref-4] Hama E, Takumi S, Ogihara Y, Murai K (2004). Pistillody is caused by alterations to the class-B MADS-box gene expression pattern in alloplasmic wheats. Planta.

[ref-5] Hollender CA, Kang CY, Darwish O, Geretz A, Matthews BF, Slovin J, Alkharouf N, Liu ZC (2014). Floral transcriptomes in woodland strawberry uncover developing receptacle and anther gene networks. Plant Physiology.

[ref-6] Ishikawa M, Ohmori Y, Tanaka W, Hirabayashi C, Murai K, Ogihara Y, Yamaguchi T, Hirano HY (2009). The spatial expression pattern of *DROOPING LEAF* orthologs suggest a conserved function in grasses. Genes, Genetic System.

[ref-7] Langfelder P, Horvath S (2008). WGCNA: an R package for weighted correlation network analysis. BMC Bioinformatics.

[ref-8] Li QF, Lu J, Yu JW, Zhang CQ, He JX, Liu QQ (2018). The brassinosteroid-regulated transcription factors BZR1/BES1 function as a coordinator in multisignal-regulated plant growth. Biochimica et Biophysica Acta-Gene Regulatory Mechanisms.

[ref-9] Li Z, Liu D, Xia Y, Li ZL, Jing DD, Du JJ, Niu N, Ma SC, Wang JW, Song YL, Yang ZQ, Zhang GS (2020). Identification of the WUSCHEL-related homeobox (*WOX*) gene family, and interaction and functional analysis of *TaWOX9* and *TaWUS* in wheat. International Journal of Molecular Sciences.

[ref-10] Liao ML, Peng ZS, Yang ZJ, Wei SH, Martinek P (2015). Identification of differentially expressed genes in a pistillody common wheat mutant using an annealing control primer system. Genetics and Molecular research.

[ref-11] Livak KJ, Schmittgen TD (2001). Analysis of relative gene expression data using real-time quantitative PCR and the 2^−ΔΔCt^ method. Methods.

[ref-12] Ma SS, Ding ZH, Li PH (2017). Maize network analysis revealed gene modules involved in development, nutrients utilization, metabolism, and stress response. BMC Plant Biology.

[ref-13] Marcos-Barbero EL, Perez P, Martinez-Carrasco R, Arellano JB, Morcuende R (2021). Screening for higher grain yield and biomass among sixty bread wheat genotypes grown under elevated CO_2_ and high-temperature conditions. Plants.

[ref-14] Meguro A, Takumi S, Ogihara Y, Murai K (2003). WAG, a wheat *AGAMOUS* homolog, is associated with development of pistil-like stamens in alloplasmic wheats. Sex Plant Reproduction.

[ref-15] Murai K (2013). Homeotic genes and the ABCDE model for floral organ formation in wheat. Plants.

[ref-16] Murai K, Takumi S, Koga H, Ogihara Y (2002). Pistillody, homeotic transformation of stamens into pistil-like structures, caused by nuclear-cytoplasm interaction in wheat. Plant Journal.

[ref-17] Murai K, Tsunewaki K (1993). Photoperiod-sensitive cytoplasmic male sterility in wheat with Aegilops crassa cytoplasm. Euphytica.

[ref-18] Paolacci AR, Tanzarella OA, Porceddu E, Varotto S, Ciaffi M (2007). Molecular and phylogenetic analysis of MADS-box genes of MIKC type and chromosome location of SEP-like genes in wheat (*Triticum aestivum* L.). Molecular Genetics and Genomics.

[ref-19] Patro R, Duggal G, Love MI, Irizarry RA, Kingsford C (2017). Salmon: fast and bias-aware quantification of transcript expression using dual-phase inference. Nature Methods.

[ref-20] Pautot V, Dockx J, Hamant O, Kronenberger J, Grandjean O, Jublot D, Traas J (2001). KNAT2 evidence for a link between knotted-like genes and carpel development. Plant Cell.

[ref-21] Peng ZS (2003). A new mutation in wheat producing three pistils in a floret. Journal of Agronomy and Crop Science.

[ref-22] Peng ZS, Yang ZJ, Ouyang ZM, Yang H (2013). Characterization of a novel pistillody mutant in common wheat. Australian Journal of Crop Science.

[ref-23] Ramírez-González RH, Borrill P, Lang D, Harrington SA, Brinton J, Venturini L, Davey M, Jacobs J, Van Ex F, Pasha A, Khedikar Y, Robinson SJ, Cory AT, Florio T, Concia L, Juery C, Schoonbeek H, Steuernagel B, Xiang D, Ridout CJ, Chalhoub B, Mayer KFX, Benhamed M, Latrasse D, Bendahmane A, Wulff BBH, Appels R, Tiwari V, Datla R, Choulet F, Pozniak CJ, Provart NJ, Sharpe AG, Paux E, Spannagl M, Bräutigam A, Uauyet C (2018). The transcriptional landscape of polyploid wheat. Science.

[ref-24] Roberts WR, Roalson EH (2017). Comparative transcriptome analyses of flower development in four species of Achimenes (Gesneriaceae). BMC Genomics.

[ref-25] Saraike T, Shitsukawa N, Yamamoto Y, Hagita H, Iwasaki Y, Takumi S, Murai K (2007). Identification of a protein kinase gene associated with pistillody, homeotic transformation of stamens into pistil-like structures, in alloplasmic wheat. Planta.

[ref-26] Shannon P, Markie A, Ozier O, Baliga NS, Wang JT, Ramage D, Amin N, Schwikowski B, Ideker T (2003). Cytoscape: a software environment for integrated models of biomolecular interaction networks. Genome Research.

[ref-27] Stelzl U, Worm U, Lalowski M, Haenig C, Brembeck FH, Goehler H, Stroedicke M, Zenkner M, Schoenherr A, Koeppen S, Timm J, Mintzlaff S, Abraham C, Bock N, Kietzmann S, Goedde A, Toksöz E, Droege A, Krobitsch S, Korn B, Birchmeier W, Lehrach H, Wanker EE (2005). A human protein-protein interaction network: aresource for annotating the proteome. Cell.

[ref-28] Su YL, Liu JX, Liang WQ, Dou YH, Fu RF, Li W, Feng C, Gao C, Zhang D, Kang Z, Liet H (2019). Wheat AGAMOUS LIKE 6 transcription factors function in stamen development by regulating the expression of *Ta APETALA3*. Development.

[ref-29] Sun QX, Qu JP, Yu Y, Yang ZJ, Wei SH, Wu YL, Yang J, Peng ZS (2019). TaEPFL1, an EPIDERMAL PATTERNING FACTOR-LIKE (EPFL) secreted peptide gene, is required for stamen development in wheat. Genetica.

[ref-30] Tian T, Liu Y, Yan HY, You Q, Yi X, Du Z, Xu WY, Su Z (2017). agriGO v2.0: a GO analysis toolkit for the agricultural community, 2017 update. Nucleic Acids Research.

[ref-31] The International Wheat Genome Sequencing Consortium (IWGSC) (2014). A chromosome-based draft sequence of the hexaploid bread wheat (Triticum aestivum) genome. Science.

[ref-32] Ullah A, Nadeem F, Nawaz A, Siddique KH, Farooq M (2022). Heat stress effects on the reproductive physiology and yield of wheat. Journal of Agronomy and Crop Science.

[ref-33] Wang XL, Kong HZ, Hong MF (2009). F-box proteins regulate ethylene signaling and more. Genes Development.

[ref-34] Wang QH, Yang ZJ, Wei SH, Jiang ZY, Yang YF, Hu ZS, Sun QX, Peng ZS (2015). Molecular cloning, characterization and expression analysis of *WAG-1* in the pistillody line of common wheat. Genetics Molecular Research.

[ref-35] Yamamoto M, Shitsukawa N, Yamad M, Kato K, Takumi S, Kawaura K, Ogihara Y, Murai K (2013). Identification of a novel homolog for a calmodulin-binding protein that is upregulated in alloplasmic wheat showing pistillody. Planta.

[ref-36] Yang Q, Yang Z, Tang H, Yu Y, Chen Z, Wei S, Peng Z (2018). High-density genetic map construction and mapping of the homologous transformation sterility gene (*hts*) in wheat using GBS markers. BMC Plant Biology.

[ref-37] Yang W, Lou X, Li J, Pu M, Mirbahar AA, Liu D, Sun J, Zhan K, He L, Zhang A (2017). Cloning and functional analysis of MADS-box genes, TaAG-A and *TaAG-B*, from a wheat K-type cytoplasmic male sterile line. Frontiers in Plant Science.

[ref-38] Yang ZJ, Peng ZS, Wei SH, Liao ML, Yu Y, Jang ZY (2015). Pistillody mutant reveals key insights into stamen and pistil development in wheat (*Triticum aestivum* L.). BMC Genomics.

[ref-39] Zhang B, Horvath S (2005). A general framework for weighted gene co-expression network analysis. Statistical Applications in Genetics and Molecular Biology.

[ref-40] Zhang L, Yang ZJ, Peng ZS, Yu Y, Sun QX (2015). Molecular cloning, characterization, and expression analysis of DROOPING LEAF gene in the pistillody line of common Wheat. Modern Applied Science.

[ref-41] Zhu XX, Li QY, Shen CC, Duan ZB, Yu DY, Niu JS, Ni YJ, Jiang YM (2015). Transcriptome analysis for abnormal spike development of the wheat mutant dms. PLOS ONE.

[ref-42] Zhu Y, Saraike T, Yamamoto Y, Hagita H, Takumi S, Murai K (2008). orf260cra, a novel mitochondrial gene, is associated with the homeotic transformation of stamen into pistil-like structures (pistillody) in alloplasmic wheat. Plant Cell Physiogy.

